# Development of an innovative measurement method for patellar tracking disorder

**DOI:** 10.18632/aging.202161

**Published:** 2020-12-01

**Authors:** Zhe Xue, Zheng Pei, Hui Zhang, Chong Tang, Junxiu Jia, Kun Zhang, Keshi Zhang, Zhenpeng Guan

**Affiliations:** 1 Department of Orthopedics, Peking University Shougang Hospital, ShiJingshan 100144, Beijing, P.R. China

**Keywords:** patellar tracking, measurement method, patellofemoral joint instability, patellar dislocation

## Abstract

In this study, we investigated whether the measurement of patellar tracking can be used as a diagnostic parameter of patellofemoral joint disease. Patellar tracking is defined as the movement of the patella in relation to the femorotibial joint within the full range of flexion and extension of the knee joint. The PubMed, EMBASE, Medline, PsychINFO, and AMED databases were used to find relevant articles. Analyzed were the patellar tracking coordinate system and the measurement objects, precision, methods used in those studies, as well as the results obtained. Origin points for coordinate systems varied across the studies. The research object and methods of patellar tracking varied in the studies. Most studies focused on a static description of the internal and external displacement and the internal and external inclination. The *in vivo*, noninvasive, and six degrees of freedom evaluation of patellar tracking reflect patellar motion more comprehensively, though each of these methods does so in different ways. Dynamic and quantitative evaluation of patellar tracking is still lacking in clinical work. Accurate and quantitative patellar tracking measurement could provide clinicians with a comprehensive evaluation of the stability of the knee joint.

## INTRODUCTION

A considerable number of patients who suffer from knee pain and instability have abnormal patellar tracking [1]. Knee-joint movement is accomplished through a complex system, with the knee extension device at the center, involving the bone structure and the surrounding muscle and soft tissue [2, 3]. Patellar tracking is the movement of the patella relative to the femorotibial joint within the full range of flexion and extension of the knee joint [4]. If the patella moves abnormally, then patellofemoral joint is unstable, which causes pain [3].

The etiological mechanism of patellar maltracking is still unclear. There are four main pathological mechanisms [5]: (1) abnormalities of the muscles and soft tissues around the knee extension device, including the dynamic structure, such as the morphological abnormalities of the quadriceps [6]; (2) static structural abnormalities, such as a medial patellofemoral ligament injury, severe lateral structure tightness [7], and patella alta [8]; (3) abnormal bony morphology [9], such as increased Q angle, knee valgus, knee hyperextension, and patellar morphology [9, 10]; and (4) abnormal morphology of the external femoral condyle caused by degenerative deformation or dysplasia [11].

Research studies have focused on the patellar tracking, varying from one to six degrees of freedom of motion, in healthy people [8] versus the populations with patellofemoral joint pain [12] and patellar instability [13] using clinical and cadaveric studies. Methods used in these studies were X-ray, computed tomography [11], nuclear magnetic resonance imaging [1], infrared ray capture system [14], electromagnetic capture system [3], and holographic camera capture methods [15]. The studies found that patellar tracking was different in patients with patellofemoral pain and instability than in healthy people. Because previous studies mainly focused on qualitative or semi-quantitative explanations, there was a lack of quantitative and dynamic analyses, leaving the use of patellar tracking as a method for assessing pain or instability of the patellofemoral joint up for debate.

This paper introduces the progress of patellar tracking research, specifically the definition and naming of the coordinate system, measurement objects, measurement methods, and measurement results.

## RESULTS

### Definition and naming of coordinate system

**Figure 1:**
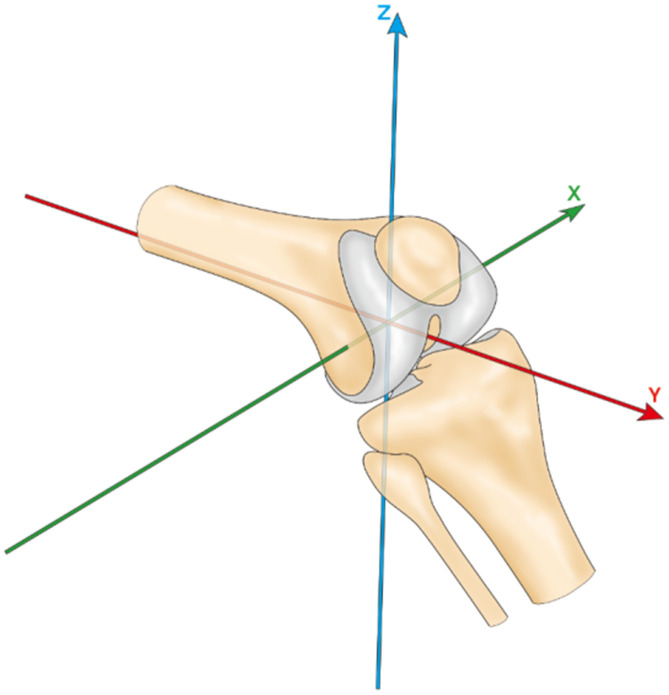
**Knee coordinate system.**

**Figure 2:**
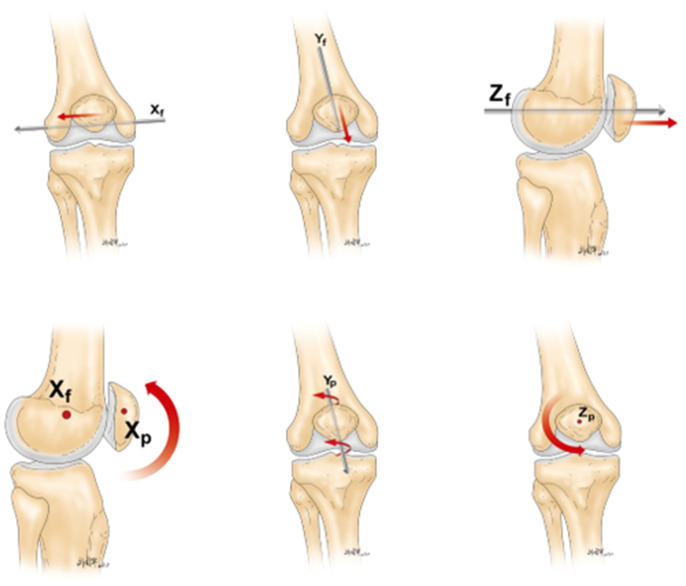
**Six degrees of freedom movement of the patella relative to the femorotibial joint.**

The studies analyzed for this paper used different origin of coordinates in their research. O'Donnell [1] took the lowest point of the femoral trochlear as the origin of the coordinate system. Reider [5] took the tibial tubercle as the reference point. Nha [15] took the midpoint of the connection between the internal and external epicondyle of femur as the reference point. Lin [8, 16] and Amis et al. [3] took the midpoint of the connection between the posterior femoral condyles as the reference point (Table 1). Lin and Amis et al. set up the space rectangular coordinate system using the midpoint between the posterior femoral condyles as the origin point (translated to the center of femoral shaft axis) (Figure 1). They also defined three vertical axes of translational verse and six-dimensional rotational symmetry of the patellar tracking respectively relative to the coordinate system, including non-rotational displacement along the X, Y, and Z axes. The rotational motions around the three axes are flexion and extension rotation, lateral and medial inclination, and lateral and medial rotation, respectively (Figure 2). The joint coordinate system should describe the patellar tracking relative to the femorotibial joint so there is uniformity among studies.

**Table 1:** The origin of knee coordinate system.

**Study**	**Orgin**
Kita K [18]	deepest point of trochlear groove
Tanaka MJ [19]	deepest point of trochlear groove
Reider B [5]	tibial tuberosity
Lin [16]	midpoint of the posterior
	femoral condyles
Lin [8]	midpoint of the posterior
	femoral condyles
O'Donnell P [1]	midpoint of the posterior
	femoral condyles
Nha KW [15]	midpoint of the posterior
	femoral condyles
Wilson NA [14]	midpoint of the lateral-posterior
	femoral condyles

### Measurement objects

The literature states the patellar tracking research objects were generally divided into cadaveric and clinical studies, with different accuracies and active/passive flexions and extensions, as well as advantages and disadvantages (Table 2 and Table 3). The mentioned advantages of cadaveric research included easy sampling, strong operability, no ethical problems, and the possibility of adding artificial intervention. However, the accuracy was lower than that of clinical research and it was a non-physiological microenvironment. The aforementioned shortcomings could be overcome in clinical studies; however, most of the early clinical studies were invasive operations [10, 17], which required skilled operators and a high-level of patient’s cooperation.

**Table 2:** Accuracy of active and passive flexion and extension of knee in cadaveric study.

**Researcher**	**Object**	**Accuracy**	**Active or Passive**
Amis [3]	8 men bodies (8knees, Right)	translation <0.3mm, rotation <0.5°	Passive
Reider.B [5]	20 men bodies	translation <0.5mm	Passive

**Table 3:** Accuracy of active and passive flexion and extension of knee *in vivo*.

**Researcher**	**Object**	**Accuracy**	**Active or Passive**
Fang Lin [8]	12 healthy people (18 knees)	not mentioned	active
Fang Lin [16]	7 healthy women; 9 AKP patients	not mentioned	active
Donnell [1]	50 healthy people (97 knees)	not mentioned	active
Wilson NA [14]	10 healthy people	translation <1.1mm, rotation <1.2°	active
Nha KW [15]	8 healthy people	translation <0.001 mm, rotation < 0.3°	active
Kita K [18]	24 RPD patients (25 knees)	not mentioned	Passive
Carlson VR [12]	12 RPD patients (20 knees); 13 healthy people (20 knees)	translation <0.3mm	active
Tanaka.MJ [19]	38 RPD patients (76 knees)	translation <0.1mm, rotation <0.1°	active

AKP: anterior knee pain; RPD: recurrent patellar dislocation.

In previous studies, methods used to measure patellar tracking included computed tomography, nuclear magnetic resonance imaging, an infrared tracking system, and a fluorescence capture system to chase the patellar tracking. This has shown there is variation in the knee’s range of motion (Table 4).

**Table 4:** Measurement methods of patellar tracking.

**Method**	**Researcher**	**Recording Conditions**	**Equipment**
CT	Guzzanti V [11]	Fixed at 150° classified into 4 grades	Siemens Aktiengesellschaft, Medical Engineeering Group, Erlanger, Federal Republic of Germany
DKCT (Dynamic CT)	Tanaka MJ [19]	0°-70°, record once per 10°	Toshiba America Medical Systems Corporation
MRI	Donnell [1]	30°-- 0°, continuous imaging classified into 4 grades	1.0 T unit, Siemens Impact
Dynamic MRI (CPC-MRI)	Carlson VR [12]	5°-- 45°	Cine Phase-Contrast (CPC) MRI in a 3-T MRI Scanner (Philips).
Infrared capture system	Fang Lin [8]	0°-- 20°	OPTOTRAKe 3020 Motion Capture System (Northern Digital, Inc, Waterloo, Canada
Infrared capture system	Wilson NA [14]	Range:0°-120°, record the position at the angle of 0°, 15°, 30°, 45°, 60°, 75°, 90°	OPTOTRAKe 3020 motion capture system (Northern, Digital, Inc, Waterloo, Canada
Fluorescence transmission capture system+MRI	Nha KW [15]	Lunge squat at, 0°, 30°, 60°, 75°, 90°, 105°, 120° and max flexion angle	3.0-T MRI, Scanner (Siemens)
Electromagnetic tracing system	Amis [3]	Passive flexion during 0°--100 °	Electromagnetic Tracking System(Flock of Birds;Ascension Technology, Burlington, VT)
Arthroscopy	Kita K [18]	Passive flexion during 0°--60°	Video through the anterior-lateral portal of arthroscopy

### Medial and lateral translation

The medial and lateral translation of the patellar tracking during the flexion and extension were different in healthy people. However, except for the micro-movement, the patella moved along a straight line. Some research studies revealed the patella translated first medially and then laterally. Amis [3] demonstrated that the patella translated medially for 5 mm (0°-20°) first and laterally for 11.5 mm (20°-90°). In Reider's study [5], 15% of the patellas first moved medially (0°-30°) then laterally; and 85% of the patellas moved laterally the entire time. Lin [8] showed that the patella first moved medially (0°-5°) and then laterally (5°-15°). One study reported that the patella moved laterally, then medially, and then laterally again. Nha [15] demonstrated that the patella moved medially for 1.5 mm (0°-30°), then laterally for 2.2 mm (30°-90°), and again medially for 0.8mm (90°-135°). Other studies found that the patella always moved laterally. In O'Donnell’s study [1], 33% of the samples moved laterally 1/3 of the width of the longest axis of the patella, 9% of the samples moved laterally 2/3 of the width of the patella, and the rest showed no significant displacement. Carlson [12] believed that the patella always moves laterally (5°-45°). On the other hand, one researcher reported the patella moved medially all the time. Wilson [14] believed that the patella shifted medially for 7.73 mm from the range of 15° to 90° of knee flexion.

In the patients with anterolateral knee pain, the patella always moved laterally during the knee flexion to extension [1, 11, 12, 14]. In O'Donnell’s [1] study, 13% of the samples moved laterally 1/3 of the width of the patella, 17% of the samples moved laterally 2/3 of the width of the patella, and 7% of the samples moved laterally the width of the patella. Guzzanti [11] reported that the patella always moved laterally. Wilson’s study [14] demonstrated the patella moved laterally for 3.92 mm (0°-90°). Carlson [12] reported the patella moves laterally (5°-45°) through the entire circular knee movement. Cadaveric studies may show different treatments of the medial and lateral support tissue correlate with the patellar tracking pattern. If the medial support retinaculum was cut off, then the lateral displacement of the patella would increase; if the lateral support retinaculum was released, then the tracking was consistent with that of healthy people. After the medial retinaculum compression was combined with the lateral retinaculum release, the medial patellofemoral transverse displacement (0°-90°) was increased. In patients with patellar dislocation, the patella always moved laterally: Kita [18] believed that the patella always moved laterally (0°-60°).

### Medial and lateral inclination

In healthy people, some studies revealed that the patella inclined medially first and then laterally during the extension to flexion. Reider [5] reported that 15% of the patients inclined medially first (0°-30°) and then laterally, 85% of the patients inclined laterally during the whole circle of knee movement. Wilson [14] believed the patella inclined medially to -1.8° within the 0°-45° range, and inclined laterally to 2.5° within the 45°-90° range. Some studies reported that the patella firstly inclined laterally and then medially. Nha [15] demonstrated that the patella inclined laterally 3.6° within the 0°-75° range and medially 5.2° within the 75°-135° range. Guzzanti [11] reported research that the patella always inclined laterally, but angle was less than 8°. Carlson [12] believed that the patella always inclined laterally as well, but within the range of 5°~45°. However, Lin [8] similarly said that the patella inclined laterally, but about 2.4° within the 0°-15° range.

Guzzanti [11] and Wilson [14] reported that, compared with the healthy population, patients with anterior knee pain showed a greater tendency for lateral patellar inclination. Carlson [12] believed that the patella inclined internally and then externally. Reider [5] reported the release of the lateral retinaculum, or the contraction of the medial retinaculum, would reduce the trend of lateral inclination, but cutting the medial retinaculum would increase the trend of lateral inclination.

### Internal and external rotation

Reider [5] reported that in healthy people the patella always rotated internally during the knee flexion to extension. According to Nha’s study [15], the patella first rotates laterally by 1.1° (135°-120°) and then internally by 8.1° (120°-0°). Amis [3] and Carlson [12] argued that the patella has no obvious pattern to follow in terms of rotational freedom. In the studies where the focus was patients with anterior knee pain, Wilson [14] believed that the patella always rotates laterally and Carlson [12] speculated the patella rotated internally and externally.

### Flexion and extension

Conclusions on the flexion and extension of the patella were the same across studies [8, 12, 14, 15], and the patella always showed the stretching motion during the process from extension to flexion.

In patients with anterior knee pain, Wilson [14] showed that the pattern of patellar flexion and extension movement was the same as that of healthy people except that the degree of the flexion angle increased when approaching 90° of flexion.

### Proximal and distal translation

In the healthy population and in patients with anterior knee pain, the patella continued to move proximally while the knee was extended [8, 12].

### Anterior and posterior translation

There are few recorded studies in the literature on the degree of freedom of anterior and posterior translation. Lin [8] reported that, in the healthy population, the patella always had an anterior translation (15°-0°). Cardson [12] believed that patella moved anteriorly and then posteriorly.

## DISCUSSION

Our study reviewed and summarized research studies on the reference point, coordinate system, and measurement objects, methods, and results of patellar tracking.

Some studies used the tibial tubercle [5], the deepest point of femoral trochlear groove [18], and other osseous reference points, while also using the congruence angle, trochlear groove angle, and Q angle to chase the patellar tracking. Such patterns of description were known as the "osseous morphology method" [13]; however, this method depended on the osseous morphology of the objects too much. Any abnormality (such as congenital malformations) could lead to a greater bias in the measurement result. The "six degrees of freedom coordinate system" proposed by Lin et al. [8, 16] (Figure 1 and Figure 2) could weaken the dependence on bone morphology and directly record the tracking of the patella relative to the origin of coordinates, thus obtaining more objective and accurate results.

In some studies, cadaveric specimens were used to measure the patellar tracking [3, 5]. Although the operations were simple and without ethical problems, it was difficult to simulate the state of soft tissue around the knee under physiological conditions (such as quadriceps, muscle force, and ligament and soft tissue tension), resulting in a large systematic error. In recent years, researchers have performed the study *in vivo* frequently without anesthesia [8, 12, 14–16], which achieves a higher measurement accuracy and smaller error, resulting in the maximum recording the physiological condition of patellar tracking. Nevertheless, there were still some limitations, including the complex instrument operation and the high demand of cooperation between knee joint motion muscle strength of patients. No measurement method can currently be used to quantitatively evaluate the continuous dynamics of patellar tracking, and further research is still needed.

Studies reported that as the knee joint moves from flexion to extension, there are six degrees of freedom movement for the patella, but there are two main degrees of freedom within the patellar medial/lateral translation and inclination. In healthy people [1, 3, 5, 8, 12, 14, 15], the patellar moved along a straight line (some patients presented the medial and lateral micro-movement in the studies). However, in patients with patellofemoral pain and instability [1, 5, 11, 12, 14, 18], there was a significant lateral patellar translation and inclination. This suggests abnormal patellar tracking might be one of the symptoms and causes of patellofemoral pain and instability.

The mechanism of patellofemoral pain and instability has not yet been clarified. Previous surgical intervention methods focused on the rearrangement of soft tissue structures, such as medial retinaculum compression or lateral retinaculum release, to relieve the pain from the patellofemoral joint [20, 21]. The reconstruction of the medial patellofemoral ligament, the first-grade stable structure of the patella, was pivotal in the treatment of patellofemoral joint instability [22]. However, there are studies that reported there was a long-term failure rate of up to 20% for this operation [2]. Therefore, it is particularly important to correct bone deformities, such as patella alta, abnormal femoral trochlear, external tibial tubercle, excessive femoral anteversion angle, and tibial external rotation angle. Additionally, studies have shown that Caton index >1.2, type B and D femoral trochlear morphology, tibial tuberosity-trochlear groove distance >20mm [13], femoral anteversion >23°, and tibial external rotation angle >30° are possible causes of surgical failure [23, 24] and patellar maltracking [25, 26]. The excessive femoral anteversion angle could cause sudden patellar lateral translation at the extremity of the knee extension, defined as the "J sign" [4]. This may be a cause of failure as well, and we speculate that it is one of the causes of long-term medial patellofemoral ligament reconstruction failure. Therefore, a comprehensive, accurate and dynamic evaluation of the patellofemoral joint was necessary for patients with patellofemoral pain and instability.

According to the research, patellofemoral joint pain and instability is associated with early patellofemoral osteoarthritis [27]. If patellar maltracking was present in individuals from childhood, but it was not corrected in a timely manner, there would be an increase in patellofemoral joint pressure, potentially causing the cartilage injury and femoral trochlear severe deformity to progress. This could lead to an increased risk of long-term adverse outcomes and accelerate the rate of the patellofemoral joint osteoarthritis [28–30] as an adult. Studies have shown that the incidence of patellofemoral osteoarthritis within 15 years of patellar maltracking detection was as high as 50%, even after the initial patellar dislocation was corrected by surgery. This rate was higher in patients with conservative treatment [31]. Therefore, early diagnose and correction of abnormal patellar tracking is important to maintain the stability of the patellofemoral joint and to prevent or delay the occurrence of osteoarthritis of patellofemoral joint.

In conclusion, the dynamic, instantaneous, and quantitative tracking mode of the patella relative to the femorotibial joint needed further exploration. By evaluating the patellar tracking of *in vivo*, noninvasive, and six degrees of freedom, the patellar tracking could be analyzed more comprehensively. However, a dynamic and quantitative evaluation of patellar tracking was still lacking in clinical work. The measurement of patellar tracking might be a new method to diagnose patellofemoral joint disease.

## MATERIALS AND METHODS

### Retrieval methods

The studies, including the cadaveric and clinical research, were retrieved from the PubMed, EMBASE, Medline, PsychINFO, and AMED databases. The keywords included patellar tracking, measurement of patellar tracking, patellar mal-tracking, patellar dislocation.

### Data collection

The collected articles were summarized from the abstract and selected according to inclusion criteria. The initial inclusion criteria included research that had: (1) at least one method describing the patellar tracking; (2) an analyzation of patellar tracking; (3) clinical or cadaveric studies; and (4) the evidence level of literature varied from I to IV. Literature that did not meet this inclusion criteria would be excluded. After the preliminary screening with this criteria, 561 references were selected as potentially related to this study. After reviewing the titles and abstracts of the articles, 57 references were selected. After excluding low-evidence-based manuscripts, reviews, and non-English research, 42 references were selected. Lastly, research that lacked a patellar tracking assessment method and measurement results were excluded. After the last exclusions, 19 research manuscripts were selected and used for this paper’s analysis.
